# Comparison of the Effects of Postoperative Arm Restraints and Mittens on Cleft Lip Scar Quality after Primary Repair

**DOI:** 10.3390/jcm13133619

**Published:** 2024-06-21

**Authors:** Alexandra N. Verzella, Matteo Laspro, Allison Diaz, Michael F. Cassidy, Jenn Park, Jill Schechter, Andre Alcon, Pradip R. Shetye, David A. Staffenberg, Roberto L. Flores

**Affiliations:** Hansjörg Wyss Department of Plastic Surgery, New York University Grossman School of Medicine, New York, NY 10016, USA; alexandra.verzella@nyulangone.org (A.N.V.); matteo.laspro@nyulangone.org (M.L.); allison.diaz@nyulangone.org (A.D.); michaelfcassidy3@gmail.com (M.F.C.); jenn.park@nyulangone.org (J.P.); jwm2161@cumc.columbia.edu (J.S.); andre.w.alcon@kp.org (A.A.); pradip.shetye@nyulangone.org (P.R.S.); david.staffenberg@nyulangone.org (D.A.S.)

**Keywords:** scar quality, primary cleft lip repair, arm restraints, mittens, cleft lip

## Abstract

**Introduction**: Postoperative management following primary cleft lip repair varies across institutions, cleft care teams, and individual surgeons. Postoperative precautions employed after cleft lip repair include dietary restrictions, pacifier limitations, and immobilization, with arm restraints long being used. Yet, restraint distress has led to the exploration of other forms of immobilization. Thus, this study aims to assess cleft lip scar quality and complication rates after postoperative immobilization with arm restraints versus hand mittens. **Methods**: A retrospective review of patients with unilateral cleft who underwent primary repair with the senior surgeon was done. Data on demographics, surgical characteristics, and immobilization utilized were gathered. A survey with pictures of postoperative scars were sent to laypeople who assessed scar quality with Modified Scar-Rating Scale scores for surface appearance, height, and color of the scar tissue. Statistical analysis was carried out for significance. **Results**: Twenty-eight patients with a unilateral cleft underwent arm restraints after primary lip repair, and twenty-seven utilized mittens. In total, 42 medical students completed the scar assessment. Photographs were taken an average of 23.9 (±5.8) and 28.2 (±11.9) months postoperatively in the restraint and mitten groups, respectively (*p* = 0.239). There were no statistically significant differences in scores between scar surface, height, color, or overall scar appearance. Complication rates were also similar between groups. **Conclusions**: Arm restraints appear to have no additional benefit relative to scar quality, as compared to mittens. Considering the arm restraints’ burden of care, mittens should be considered as a measure to protect the lip after primary repair.

## 1. Introduction

Postoperative management following primary cleft lip repair is variable across institutions, cleft care teams, and individual surgeons [1]. These protocols of care are guided by the desire to protect the lip reconstruction as it heals but are largely based on individual or institutional experience rather than evidence-based approaches. Indeed, while it is generally accepted that patients should undergo cleft lip repair between 3 to 6 months of age, significant variations in postoperative management still exist [2,3,4].

Postoperative precautions employed after cleft lip repair include dietary restrictions, bottle/cup and pacifier limitations, and various forms of immobilization [1]. Arm restraints have long been used for postoperative immobilization in the primary cleft lip repair population. These typically involve padded immobilizers which wrap around the upper extremities from the wrist to the axilla. The arm restraints prevent elbow flexion and, therefore, prevent the hands from touching the mouth. Up to 85% of cleft surgeons employ arm restraints to mitigate damage that infants may inflict by traumatizing the upper lip incision site with their hands [5,6,7,8]. However, cleft specialists have long debated the necessity of these restraints, and treatment recommendations differ on this subject [9,10]. Moreover, studies have shown that there are no differences in complication rates among patients who receive restraints in the postoperative period and those who do not [5,6]. Despite these findings, arm restraints are still employed by the majority of US cleft surgeons [1]. The use of restraints causes distress through inhibiting the ability of the patient to self-soothe during a time associated with pain, difficulty with feeding, and facial swelling. Mitigating the distress incurred by infants and caregivers should be balanced by the benefit to scar quality offered by arm restraints [11]. However quantitative assessments of scar quality with and without the use of arm restraints are lacking.

This study compares the effects of arm restraints and mittens on scar appearance and quality and assesses the impact of these postoperative measures on complications. We hypothesize that there are no differences in postoperative outcomes or scar quality evident between patients managed with arm restraints and patients managed with hand mittens.

## 2. Methods of the Study

### 2.1. Patient Cohort

Consistent with Internal Review Board approval (IRB#s23-01204), a single-institution, retrospective review was performed including all patients who underwent primary unilateral cleft lip repair by a single surgeon between 2017 to 2021. Inclusion criteria encompassed any patient operated on by the senior surgeons for primary unilateral cleft lip during the delineated period. This study included 28 consecutive patients who had arm restraints (Pedi-Wrap, Medi Kid Co., Norwalk, CA, USA) after their cleft lip repair (prior to December 2018), and 27 consecutive patients who underwent their cleft lip repair after the institutional transition to mittens (Protective Comfies, Hand Socks^TM^, VA, USA). Patients with postoperative photos less than one year after surgery were excluded.

### 2.2. Surgical Technique

All study patients underwent an Extended Mohler cleft lip repair by the same single surgeon. No modifications on the lip repair technique were made during the study period. All other postoperative precautionary measures remained consistent during the study period.

### 2.3. Photograph Evaluation Protocol

Postoperative photographs from the 55 patients who underwent primary unilateral cleft lip repair were de-identified, showing only the area from the apex of the nose to the upper lip. The de-identified photographs were randomly presented in an online survey using Qualtrics software (version 12/2023, Provo, UT, USA, The Modified Scar Scale developed by Mecott et al., a validated tool for scar assessment by photographs, was chosen as the means of evaluation [12]. For each photograph, participants were prompted to assign a rating of 1, 2, 3, or 4, with 1 indicating a close resemblance to normal skin, and 4 being the most extreme difference. This scoring system was used for 3 different parameters: scar surface appearance, scar height, and color mismatch. All participants were given the Modified Scar Scale teaching chart, with sample photographs and descriptions for each score and parameter, as a reference [12]. The teaching chart was accessible at all times during the survey. The anonymous online survey was sent via email to all medical students attending a single institution. Forty-two medical students completed and submitted the anonymous survey. The scores for each parameter were stratified by restraint type. A total score was generated from the sum of the scar parameters for each of the 55 photographs, ranging from 3 to 12 for each subject. A lower score designated higher scar quality.

### 2.4. Scar Scoring Parameters

For scar surface appearance, a score of 1 indicates that the scar surface appears similar to normal skin; 2 indicates slight mismatch and may be either rougher or smoother than normal skin; 3 indicates noticeably rougher-than-normal skin and may include shallow depressions or irregularities within the scar, and 4 indicates that the scar surface appears very rough compared to normal skin.

For scar height, a score of 1 indicates that a scar surface is on the same plane as normal skin; 2 indicates a smooth slope at the edge of the scar; 3 indicates a moderate and more defined slope at the edge of the scar, and 4 indicates a more extreme difference in which there is abrupt dropping at the edge of the scar.

For scar color, a score of 1 indicates minimal to no color difference; 2 indicates a subtle but noticeable difference and can include differences in pigmentation or erythema; 3 indicates a color difference that is easily distinguishable; and 4 indicates a prominent color mismatch compared to normal skin.

### 2.5. Data Collection

Utilizing the electronic medical record, demographics were collected from patients, including sex, race, age at primary surgery, and age when preoperative and postoperative photographs were taken. Variables related to the cleft lip repair included laterality of repair, presence of cleft alveolus, presence of cleft palate, cleft type, name of procedure technique, concurrent procedures, use of nasoalveolar molding (NAM), postoperative complications, 30-day readmission, and 30-day reoperations.

### 2.6. Statistical Analysis

Power analysis for a two-sample *t*-test was conducted in G*Power (Heinrich Heine Universität, Düsseldorf, Germany) to determine sufficient sample size, utilizing an α of 0.05, a power of 0.8, and an effect size of 0.5. A Chi-square test was used to compare demographic categorical variables between groups. A Fisher’s exact test was used to compare categorical variables related to the cleft lip operation. Likert score variables were analyzed via Mann–Whitney U tests and reported as medians with interquartile ranges (IQR). All *p* values of less than 0.05 were considered statistically significant.

## 3. Results

### 3.1. Patient Demographics

A total of 55 patients undergoing primary cleft lip repair were included in this study. In sum, 27 (49%) patients received arm restraints postoperatively, and 28 patients (51%) received mittens postoperatively. Patients who underwent the arm restraints protocol post-surgery were on average 3.8 (±0.7) months old, and patients who wore mittens were 3.5 (±0.7) months old (*p* = 0.041) (Table 1). Postoperative photographs were taken at 23.9 (±5.8) and 28.9 (±11.1) months in mittens and restraint groups, respectively (*p* = 0.239). A mean of 20.4 (±5.9) and 25.10 (±11.3) months elapsed between surgery and postoperative photograph for the mittens and arm restraint cohorts (*p* = 0.317). There were no statistical differences in sex or race composition between the groups (*p* = 0.121, *p* = 0.906).

### 3.2. Preoperative Diagnoses

Regarding the cleft diagnosis at time of presentation, seventeen (60.7%) of the patients who underwent the unrestrained protocol and twenty-three (85.2%) patients from the restrained protocol had a complete cleft. Twenty-two (78.5%) and twenty-five (92.6%) patients from the mittens and arm restraints groups, respectively, presented with a cleft alveolus. Furthermore, sixteen (57.1%) and nineteen (70.4%) of the patients from the mittens and arm restraints group, respectively, presented with a cleft palate. Incidences of cleft type, cleft alveolus, and cleft palate were not statistically different between the two groups (*p* > 0.05). Prior to surgery, 18 (64.3%) patients from the mittens protocol group and 24 (88.9%) patients from the restraints protocol group underwent pre-operative nasoalveolar molding (NAM), a nonsignificant difference (*p* > 0.05).

### 3.3. Concurrent Procedures

At time of surgery, all patients received the Extended Mohler lip repair with a concurrent primary cleft rhinoplasty. A total of 13 (46.4%) patients with mittens and 14 (51.9%) of patients with arm restraints also received a gingivoperiosteoplasty. There was no difference in the rates of gingivoperiosteoplasty between the groups (*p* = 0.790). Within the mittens protocol group, 4 patients received a concurrent botox injection to the upper lip. Other concurrent procedures within the mittens population included one right inguinal repair, one alveolar reconstruction, and one bilateral myringotomy and tube placement. Regarding the patients within the arm restraints group, concurrent procedures included one botox injection to the upper lip, one tongue-tie release, and an extraction of 2 teeth.

### 3.4. Postoperative Clinical Outcomes

Postoperative complications, wound complications, 30-day readmissions, and 30-day reoperation rates were not statistically different between groups (*p* > 0.05) (Table 2). Within the arm restraints group, one patient had a postoperative complication that led to a readmission within 30 days, which was reported in their electronic medical records as wheezing attributed to an upper respiratory infection. There were no wound complications and no 30-day reoperations reported for either group.

### 3.5. Scar Scores

**Figure 1:**
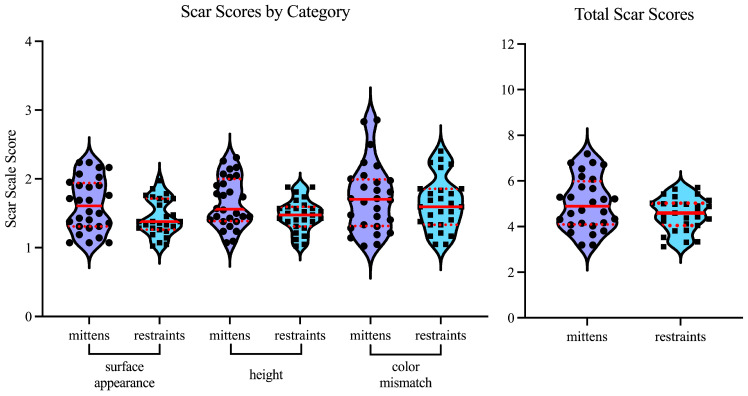
Scar scale scores, by category and overall, for mittens and restraints cohorts. Solid red lines indicate the median response. Red dotted lines represent interquartile ranges. Points indicate the average score for each individual patient.

Forty-two medical students assigned a score from 1–4 for each parameter on the modified scar scale for each patient in our study. Scores were analyzed by protocol group (mittens vs. restraints), and the average score for each patient in each category was calculated. The average categorical scores for each patient in the study were then used for analysis. For scar appearance, the median score in the no-restraints group was 1.61 (1.31–1.94), versus 1.38 (1.26–1.71) in the restraints group (Table 3, Figure 1). For scar height, the median score in the mittens group was 1.56 (1.39–2.00), versus 1.48 (1.31–1.60) in the restraints group. For scar color, the median score in the mittens group was 1.70 (1.32–1.99), compared to 1.60 (1.33–1.86) in the restraints group. While the median score for the restraints group was lower in all categories, this difference was not found to be statistically significant (appearance *p* = 0.067, height *p* = 0.068, and color mismatch *p* = 0.631). Moreover, the difference in total scores was not significantly different between the two treatment cohorts (*p* = 0.298).

## 4. Discussion

This study found that the use of mittens instead of arm restraints post-primary cleft lip repair does not affect complications and aesthetic scar outcomes. Only one postoperative complication leading to readmission within 30 days was noted from the arm restraints group, and these rates were not statistically different from those in the mittens group (*p* value > 0.05). Notably, the reason for readmission appears to be independent of the use of arm restraints. No wound complications, 30-day reoperations, or other postoperative complications were noted in the study patients.

The debate about whether arm restraints are beneficial after cleft surgery in infants is not a new one. An early publication from the United Kingdom reported that performing lip repair without the use of postoperative restraints was not associated with an increased incidence of wound trauma or breakdown [13]. Multiple subsequent studies have demonstrated that the use of restraints in the postoperative period is not necessary for the mitigation of complications [5,6,14,15]. Through analysis of video recordings of eight infants after unilateral and bilateral CL and CLP repair, a study in Japan by Tokioka et al. demonstrated that, if the repair is performed before the age of 3 to 4 months, restraints are not necessary [15]. The study found that if the infants did touch the wound site, it was without any scratching or pinching of the wound or sutures, which are movements that restraints are meant to prevent, indicating that the restraints might not be necessary, as infants were not found to be prone to these movements [15]. Importantly, the investigators reported that these infants did not have penchants for self-soothing through sucking their thumbs, an action which has previously been reported to be a “nightmare” for surgeons in this postoperative period [14]. In another study where arm restraint and no-restraint cohorts were matched for thumb sucking (*p* = 0.76), there was also no difference in complication rates between the two groups, suggesting that this action may not be as injurious as previously believed [14]. In accordance with the literature, our findings suggest no differences in postoperative complications between patients who were given arm restraints and those given mittens following cleft lip repair [5,6].

The negative impacts of arm restraints on both patients and their families have been well described [5,14]. It has been posited that restraints can induce motor weakness in infants [16,17]. Furthermore, prior reports have observed that these restraints can cause distress to the patients and their caregivers [13,14].

Given the observed distress and lack of evidence to support the use of arm restraints, the authors’ cleft unit modified their primary cheiloplasty postoperative protocol from the use of arm restraints to the use of mittens in 2018. Unfortunately, evidence-based guidelines for the postoperative management of patients with a cleft after primary lip repair are lacking, and there is a high variance in postoperative protocols [1,18]. This variance in postoperative protocols underscores the lack of standardization in the field and highlights the fact that postoperative precautionary measures are highly surgeon-specific. A systematic review performed by Ranzar et al. aimed to establish evidence-based guidelines for cleft lip perioperative management from the available literature [7]. They found that factors such as low rates of complications, surgeon preferences, selection biases, and the inability to blind to a visible intervention make high-quality evidence difficult to obtain in cleft surgery [7]. They also found that establishing guidelines is further complicated by the significant risk of bias in the current literature [7]. This study seeks to quantitatively compare the effects of arm restraints and hand mittens as to upper-lip scar quality.

Although prior studies have reported on the benefits and disadvantages of postoperative restraints as to complication rates, there is a paucity of research dedicated to evaluating the long-term scar appearance of these patients [5,6,7]. The aesthetics of pediatric facial scarring is a well-studied area, with reports detailing five main spheres negatively impacted by noticeable scarring, including physical functioning and comfort, self-esteem, social functioning and acceptability, self-confidence, and emotional stability and well-being [19]. Secondary surgery, in the form of scar revision, has been implicated in increased anxiety and depression among pediatric patients [20]. Therefore, we sought to quantify and evaluate this parameter in order to determine if mittens were non-inferior to arm restraints in terms of surgical aesthetic outcomes.

While there is no uniform or ideal method for grading in-person or photographic scar quality, multiple scales have been developed for this purpose. Previously, Eisenmann et al. compared the columellar and lip portions of unilateral cleft lip scars through three different scales: the Manchester Scar Scale (MSS), the Stony Brook Scar Evaluation Scale (SBSES), and the Modified Scar-Rating Scale (MSRS) [21]. Among the scales used in that study, the SBSES and MSS had five components, respectively, while the MSRS scale was limited to three categories, making this scale the most simple and suited for use among the layperson population [12]. Moreover, this scale was modified from the in-clinic scar scale described by Yeong et al., as it demonstrated the highest reliability when compared to other in-clinic scar scales [12,22]. Comparison and evaluation of this new scale remains a challenge, because the most prominent scales in use, like the Vancouver scar scale, are clinic-based and require the assessment of factors such as pliability, which are impossible to determine through a photography-based evaluation [23]. The main advantages of the MSRS scale, therefore, are its reliability and simplicity [12,21]. When analyzing the scores assigned for scar appearance, scar height, and scar color mismatch, our study found no statistical differences in the mean scores for each parameter, or for the total scar score, across mittens and restraint patients. The mean scores across all three parameters were between a 1 and a 2, indicating that scars in this cohort of patients were perceived as being very similar to normal skin or with a slight or subtle difference from normal skin. The similar scar quality observed in patients undergoing both mitten and arm restraint precautions, combined with the decreased distress associated with mittens when compared to restraints, would make mittens a more compelling option for postoperative cleft care.

While this study was sufficiently powered to identify that there were no significant differences between the overall scar appearances of the two cohorts, this study may be underpowered as to the identification of differences in postoperative complications, given their scarcity. Although specific elements in scar quality did approach statistical significance, suggesting that a larger-scale study could potentially find differences between the groups, it is notable that the total scar assessment score did not approach statistical significance. All patients were from a single institution, and all procedures were performed by a single surgeon, increasing the internal validity of the data, while negatively impacting the external validity of our findings. Ideally, all evaluators would be able to visualize and examine the scar in person, but this is not logistically possible, especially given that the layperson’s opinion was the one investigated in this study. It should be noted that these data may not be transferrable to patients with a bilateral cleft.

## 5. Conclusions

The replacement of arm restraints by mittens following primary cleft lip repair appears to have no negative impact on scar quality or postoperative complications. Hand mittens may be a useful strategy for protecting the upper lip after primary cleft repair, while mitigating postoperative distress experienced by the patient and the caregiver.

## Figures and Tables

**Table 1:** Patient demographics.

	Mittens	Restraints	*p* Value
	N = 28	N = 27	
	n (%)	n (%)	
Sex			0.121
Male	22 (78.6)	16 (56.3)	
Female	6 (21.4)	11(40.7)	
Race and ethnicity			0.906
White	12 (42.9)	12 (44.4)	
Asian	4 (14.3)	3 (11.1)	
Black	3 (10.7)	3 (11.1)	
Other/not specified	3 (10.7)	5 (18.5)	
Hispanic	6 (14.3)	4 (14.8)	
Age at primary surgery	3.5 (±0.7)	3.8 (±0.7)	0.041
months (±SD)			
Age at photograph	23.9 (±5.8)	28.9 (±11.1)	0.239
months (±SD)			
Time between surgery and photograph	20.4 (±5.9)	25.10 (±11.3)	0.317
months (±SD)			

**Table 2:** Preoperative diagnosis, procedure type, concurrent procedures, and postoperative complications.

	Mittens	Restraints	*p* Value
	N = 28	N = 27	
	n (%)	n (%)	
Laterality			
Left	16 (57.1)	19 (70.3)	
Right	12 (42.9)	8 (29.7)	
Cleft type			0.0683
Complete	17 (60.7)	23 (85.2)	
Incomplete	11 (39.3)	4 (14.8)	
Cleft alveolus	22 (78.5)	25 (92.6)	0.2516
Cleft palate	16 (57.1)	19(70.4)	0.2795
Procedure name			
Extended Mohler cleft lip repair	28 (100)	29 (100)	
Concurrent procedures			0.7896
Primary cleft rhinoplasty	28 (100)	27 (100)	
Gingivoperiosteoplasty	13(46.4)	14 (51.9)	
Botox injection to upper lip	4 (14.3)	1 (3.7)	
Tongue-tie release	0 (0)	1 (3.7)	
Extraction of two teeth	0 (0)	1 (3.7)	
Right inguinal hernia repair	1 (3.7)	0 (0)	
Alveolar reconstruction	1 (3.7)	0 (0)	
Bilateral myringotomy and tubes	1 (3.7)	0 (0)	
Nasoalveolar molding (NAM)	18 (64.3)	24 (88.9)	0.055
Post-op complications			0.4909
Wound complications	0 (0)	0 (0)	
Wheezing, upper respiratory infection	0 (0)	1 (3.7)	
30-day readmissions	0 (0)	1 (3.7)	0.4909
30-day reoperations	0 (0)	0	

**Table 3:** Modified Scar-Rating Scale scores for mittens and restraints groups.

	Mittens	Restraints	*p* Value
	Median (IQR)	Median (IQR)	
	N = 28	N = 27	
Scar Surface Appearance Score	1.61 (1.31–1.94)	1.38 (1.26–1.71)	0.067
Scar Height Score	1.56 (1.39–2.00)	1.48 (1.31–1.60)	0.068
Scar Color Mismatch Score	1.70 (1.32–1.99)	1.60 (1.33–1.86)	0.631
Total Scar Appearance Score	4.89 (4.09–5.99)	4.60 (4.05–5.02)	0.198

## Data Availability

The data presented in this study are available on request from the corresponding author.
